# SUMOylation in Phytopathogen Interactions: Balancing Invasion and Resistance

**DOI:** 10.3389/fcell.2021.703795

**Published:** 2021-08-16

**Authors:** Manisha Sharma, Diana Fuertes, Jordi Perez-Gil, L. Maria Lois

**Affiliations:** ^1^Centre for Research in Agricultural Genomics, CSIC-IRTA-UAB-UB, Barcelona, Spain; ^2^Biosciences, College of Life and Environment Sciences, University of Exeter, Exeter, United Kingdom; ^3^Consejo Superior de Investigaciones Científicas, Barcelona, Spain

**Keywords:** SUMO, plant defense, effectors, virus, fungi, bacteria, crop, *Arabidopsis*

## Abstract

Plants are constantly confronted by a multitude of biotic stresses involving a myriad of pathogens. In crops, pathogen infections result in significant agronomical losses worldwide posing a threat to food security. In order to enter plant tissues and establish a successful infection, phytopathogens have to surpass several physical, and chemical defense barriers. In recent years, post-translational modification (PTM) mechanisms have emerged as key players in plant defense against pathogens. PTMs allow a highly dynamic and rapid response in front of external challenges, increasing the complexity and precision of cellular responses. In this review, we focus on the role of SUMO conjugation (SUMOylation) in plant immunity against fungi, bacteria, and viruses. In plants, SUMO regulates multiple biological processes, ranging from development to responses arising from environmental challenges. During pathogen attack, SUMO not only modulates the activity of plant defense components, but also serves as a target of pathogen effectors, highlighting its broad role in plant immunity. Here, we summarize known pathogenic strategies targeting plant SUMOylation and, the plant SUMO conjugates involved in host-pathogen interactions. We also provide a catalog of candidate SUMO conjugates according to their role in defense responses. Finally, we discuss the complex role of SUMO in plant defense, focusing on key biological and experimental aspects that contribute to some controversial conclusions, and the opportunities for improving agricultural productivity by engineering SUMOylation in crop species.

## Introduction

Plant damage caused by other living organisms such as bacteria, fungi, insects, nematodes, and viruses that compromises plant growth is termed biotic stress. The pathogen infections on crops result in significant agronomic losses worldwide. Estimations point to yield losses of the five major crops (wheat, rice, maize, potato, and soybean) ranging between 17 and 30% of production, depending on the crop and the pathogen (Savary et al., 2019). Globalization, trade, and climate change, as well as increased vulnerability in production systems due to intense monoculture, have increased transboundary plant pests, threatening food and nutrition security, particularly in Africa, the Near East, and Asia (FAO, 2017).

In order to enter plant tissues, phytopathogens have to surpass physical barriers, then disable the innate immune response to obtain nutrients for propagation, and proceed to complete their infection cycle before disseminating to a new host. Plant resistance strategies against pathogens rely on a combination of morphological, biochemical, and molecular responses organized in multiple regulatory layers. These defense strategies involve the preformed physical and chemical barriers, which prevent pathogen entry and infection, and inducible immune responses that are triggered upon pathogen recognition (Bari and Jones, 2009). Pathogen- or Microbial-associated molecular patterns (PAMPs or MAMPs) are the molecular determinants that trigger inducible immune responses. PAMPs/MAMPs are recognized via plant cell surface–localized pattern-recognition receptors, resulting in the activation of pattern-triggered immunity (PTI; Cook et al., 2015). The pathogens that induce disease are successful in suppressing PTI by secreting effectors, such as toxins and effector proteins, into plant cells. In counter defense, these specialized pathogen virulence factors are recognized by the intracellular receptors, R proteins, causing activation of the second class of defense response known as effector-triggered immunity (ETI). The nucleotide-binding leucine-rich repeat (NLR) immune receptors constitute the most abundant R protein group (Bürger and Chory, 2019). The ETI is characteristically associated with programmed cell death known as the hypersensitive response (HR), which limits microbial spread by killing infected plant cells (Jones and Dangl, 2006).

The ability of pathogen effectors to suppress plant immunity is host-specific, leading to a scenario where only a small number of pathogens are successful in causing plant diseases (Bent and Mackey, 2007). ETI elicitation depends on pathogen lifestyle and it is effective against biotrophs. While biotrophs feed on living tissue, necrotrophs kill host tissue to feed on the remains. Hemibiotrophs alternate a biotrophic phase early during infection and a necrotrophic phase at later stages (Jones and Dangl, 2006). Plant hormones have a fundamental role in the coordination of defenses responses and growth balance. Salicylic acid (SA) and jasmonate (JA) are the major defense-related phytohormones. In general, SA is a positive regulator of immunity against biotrophic pathogens and antagonizes with JA, whereas JA activates defense responses against necrotrophs and in response to wounding. SA and JA antagonism seems to depend on the pathogen and have a spatial component. ETI induced by virulent bacteria renders susceptibility to necrotrophic pathogens at the site of the infection but does not suppresses JA-dependent defenses in systemic tissue (Spoel et al., 2007). Studies introducing higher resolution analyses determined that at the infection site, SA-JA antagonism occurs in adjacent cells. While SA-responses are activated in the central infection area, JA signaling is activated in cells around these central SA-active cells (Betsuyaku et al., 2018). Additional studies of this intricate interplay have found that, in some pathosystems, JA could be a positive regulator of ETI (Liu et al., 2016). The coordination of SA-JA cross-talk provides an explanation of how plants can deploy defense responses to multiple pathogens. Other hormones, such as ethylene (ET), abscisic acid (ABA), auxin, gibberellins, cytokinins, and brassinosteroids modulate immunity through interactions with SA and JA (Berens et al., 2017; Bürger and Chory, 2019). In consequence, pathogens have also developed infection strategies based on hormone-signaling interference or hormone production (Kazan and Lyons, 2014; Chanclud and Morel, 2016; Shen et al., 2018). At a molecular level, post-translational modifications (PTMs) of hormone signaling and defense components provide plants with additional regulatory layers that allow highly dynamic and rapid response to challenges, increasing the complexity and precision of cellular responses.

### Post-Translational Modifications in Plant Defense

Post-translational modifications, either constitutive or reversible, stand out as key players in determining the function of proteins and expanding the diversity of the cellular proteome. PTMs induce changes in protein activity, turnover, subcellular localization, and interactions with other molecules, enabling a fine-tuning of cellular responses. Plant defense mechanisms are largely steered by PTMs to activate appropriate signaling pathways against the invading pathogen. Conversely, some infection strategies rely on the delivery of pathogen effectors that target host cell PTMs.

Proteolytic processing constitutes a major irreversible PTM, which is exploited by plant cells to sense and inactivate pathogens, as well as by pathogens to disable the immune system (Pogány et al., 2015). Reversible PTMs consist of the addition of small molecules, such as phosphorylation, carboxylation, acetylation, methylation, prenylation, glycosylation, or the conjugation of small proteins, such as ubiquitin and ubiquitin-like modifiers. Among these, the most prevalent PTM in defense consists of phosphorylation and dephosphorylation events. Pathogen perception and immune signaling are modulated by phosphorylation cascades catalyzed by mitogen-activated protein kinase (MAPK), leading to a transcriptional reprogramming followed by defense responses initiation (Stulemeijer and Joosten, 2008; Withers and Dong, 2017). Another important PTM in plant defense involves the conjugation of ubiquitin (ubiquitylation) to a lysine in the target protein. Although proteasome-mediated protein degradation is the most frequent molecular consequence of ubiquitylation, the specific outcome is determined by the number of ubiquitin units that are added to the target and the type of ubiquitin branches built during polyubiquitin chain formation (Oh et al., 2018). In *Arabidopsis*, nearly 6% of the genome encodes for components of the ubiquitin-26S proteasome system (Vierstra, 2009), supporting its fundamental biological role (Azevedo et al., 2001; Yee and Goring, 2009; Sharma et al., 2013). Ubiquitin and other small proteins sharing the same structure (β-grasp folding) and mechanism of conjugation constitute the family of Ubiquitin-like (Ubl) modifiers (van der Veen and Ploegh, 2012; Vierstra, 2012). Ubiquitin and Ubls are conjugated to proteins through the sequential action of the E1-activating enzyme, an E2-conjugating enzyme and an E3 ligase. Dedicated cysteine proteases remove the modifier from the target, delivering the unmodified target and contributing to the modifier recycling (Vierstra, 2012; Cappadocia and Lima, 2018). Far from being excluding, different PTM can converge on a single target providing additional modulation degrees of the target (Skelly et al., 2016; Vu et al., 2018; Yin et al., 2019).

### The SUMO System in Plants

SUMO (Small Ubiquitin-like MOdifer) is an essential PTM belonging to the Ubl family of modifiers. In plants, SUMO regulates multiple biological processes, ranging from development to responses arising from environmental challenges. This biological versatility of plant SUMOylation offers novel opportunities for improving agricultural productivity (Castro et al., 2012; Benlloch and Lois, 2018; Rosa and Abreu, 2019).

Small Ubiquitin-like MOdifer is covalently attached to a K (lysine) residue located in the consensus sequence ψKxE/D, where ψ is a hydrophobic amino acid, in the substrate. As a reversible PTM, specific cysteine proteases remove SUMO from the substrate (Johnson, 2004; Jürgen Dohmen and Dohmen, 2004). The number of functional SUMO isoforms varies among organisms, from one single SUMO (Suppressor of mif two3 [Smt3]) in yeast, to four isoforms in humans (Cappadocia and Lima, 2018) and eight in *Arabidopsis*. However, only four SUMO isoforms (SUMO1, 2, 3, and 5) are expressed and are, therefore, considered functional (Kurepa et al., 2003; Benlloch and Lois, 2018). Most of the knowledge on plant SUMOylation has been generated from studies focused on *Arabidopsis* (Table 1). In *Arabidopsis*, SUMO paralogs display distinct conjugation and maturation properties (Chosed et al., 2006; Castaño-Miquel et al., 2011) and expression patterns (van den Burg et al., 2010), supporting the existence of a functional diversification with implications in cell physiology. *Arabidopsis* SUMO1 and SUMO2 are closely related proteins, expressed at high levels, and essential during the early stages of seed development (Saracco et al., 2007). Under standard growth conditions, SUMO1/2 accounts for most of the SUMO conjugates detected *in planta*. Abiotic stress treatments, such as heat shock, dramatically induce the formation of high molecular weight SUMO1/2 conjugates (Kurepa et al., 2003). The predominant conjugation of the essential SUMO1/2 isoforms is supported by their high-affinity interactions with the E1-activating and E2-conjugating enzymes in comparison to the non-essential SUMO3 and 5 isoforms (Castaño-Miquel et al., 2011). Initial genetic approaches based on the overexpression of either SUMO1 or SUMO2 determined a negative role of SUMOylation in ABA signaling (Lois et al., 2003), although they seem to mediate most of the biological roles of SUMO in plants. In contrast to SUMO1/2, SUMO3 biological role seems to be restricted to flowering regulation and plant defense responses (van den Burg et al., 2010; Saleh et al., 2015), while the role of SUMO5 remains to be uncovered.

**TABLE 1:** SUMO machinery components in *Arabidopsis*.

	Gene code	Name	Alternative name
*SUMO*	AT4G26840	SUM1	
	AT5G55160	SUM2	
	AT5G55170	SUM3	
	AT2G32765	SUM5	
*E1*	AT2G21470	SAE2	
	AT4G24940	SAE1a	
	AT5G50580	SAE1b	
	AT5G50680	
*E2*	AT3G57870	SCE1	AtSCE1a
*E3*	AT5G60410	SIZ1	
	AT3G15150	HPY2	AtMMS21
	AT1G08910	PIAL1	
	AT5G41580	PIAL2	
*ULP C48*	AT4G15880	ESD4	
	AT3G06910	ELS1	ULP1a
	AT4G00690	ELS2	ULP1b
	AT1G10570	OTS2	ULP1c
	AT1G60220	OTS1	ULP1d
	AT1G09730	SPF1	ASP1
	AT4G33620	SPF2	
	AT3G48480	FUG1	ULP1e
*ULP C97*	AT4G25660	Desi 1	
	AT4G25680	Desi 2A	
	AT1G47740	Desi 2B	
	AT2G25190	Desi 3B	
	AT5G25170	Desi 3C	
	AT4G17486	Desi 4A	
	AT5G47310	Desi 4B	

The SUMO proteases are cysteine proteases responsible for SUMO maturation and deconjugation from target proteins. SUMO proteases are the SUMO system component displaying the largest diversification in *Arabidopsis* (Benlloch and Lois, 2018; Castro et al., 2018; Morrell and Sadanandom, 2019), which translates into SUMO isoform specificity (Chosed et al., 2006; Colby et al., 2006). *Arabidopsis* SUMO proteases are classified into two distinct groups of cysteine proteases: the Ulp (Ubiquitin-like protease) and the Desi (deSUMOylating isopeptidase) families (Nayak and Müller, 2014). Ulps belong to the C48 subgroup of the CE superfamily characterized by a catalytic triad His-Asp-Cys, whereas Desi enzymes belong to the C97 subgroup and possess the catalytic dyad His-Cys (Hickey et al., 2012).

The E1-activating enzyme is a heterodimer composed of the SAE2 large subunit and the SAE1 small subunit. *Arabidopsis* expresses two SAE1 isoforms (SAE1a and SAE1b) that display distinct catalytic activities (Castaño-Miquel et al., 2013), suggesting a regulatory role of the E1 in SUMOylation *in planta*. The E1 is organized in four distinct functional domains, and three of them are located at the SAE2 large subunit (Lois and Lima, 2005). Recognition of SUMO by the E1 is the first committed step into SUMO conjugation (SUMOylation) and is controlled by the adenylation domain, which is located at the heterodimer interface. Subsequently, adenylated SUMO is transferred to the SAE2 catalytic cysteine establishing a high-energy thioester bond. The recruitment of the E2 by the SAE2 UFD (ubiquitin-fold domain) domain facilitates a transesterification reaction delivering an E2 loaded with SUMO. The SAE2 contains an additional domain, referred as the C-terminal domain, that is a disordered domain responsible for SUMO E1 nuclear localization (Castaño-Miquel et al., 2013; Más et al., 2020). The high specificity identified in E1-E2 interactions, most likely as a result of a coevolution process, highlight the importance of conducting biochemical studies using homologous systems to obtain robust results (Reiter et al., 2013, 2016; Liu et al., 2017; Liu B. et al., 2019). The loaded E2 is competent to catalyze SUMOylation to the protein target. *Arabidopsis* contains only one isoform of the E2-conjugating enzyme (SCE1; Vierstra, 2012). Interestingly, SCE1 levels are increased in plants engineered to have reduced endogenous SUMOylation (Saracco et al., 2007; Castaño-Miquel et al., 2017). The potential existence of an unknown mechanism to modulate SCE1 levels suggests that SCE1 could play a key role in controlling endogenous SUMOylation rate. SUMO target specificity is further provided by SUMO ligases that comprise four components, SIZ1 and MMS21/HPY2, PIAL1, and PIAL2 (Kurepa et al., 2003; Ishida et al., 2009; Tomanov et al., 2014). SIZ1 and MMS21 facilitate SUMOylation to substrates (E3 ligases), while PIAL1/2 have been proposed to promote SUMO chain formation (E4 ligases). Studies using plants with T-DNA insertions in the *SIZ1* E3 ligase revealed an important role of SUMOylation in various abiotic stress responses, including salinity, drought, heat, oxidative or nutrient deficiency, and plant immunity (Augustine and Vierstra, 2018; Verma et al., 2018).

Here, we seek to highlight the findings that have established the versatile role of SUMOylation as a key post-translational regulatory mechanism to provide rapid responses to biotic stresses, and the virulence strategy employed by pathogens to counteract the immune system by targeting SUMO machinery components of the plant.

## Pathogens Strategically Target Host SUMOylation

**FIGURE 1:**
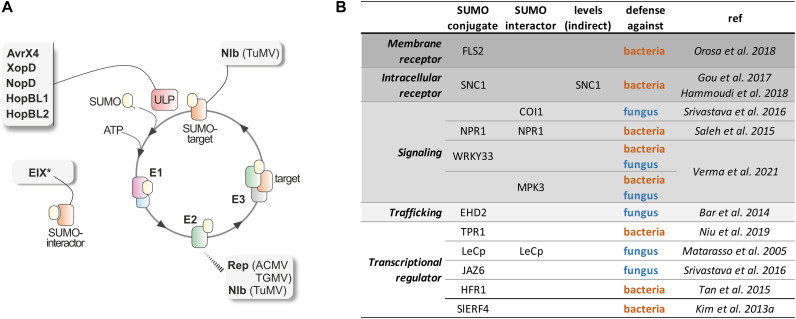
SUMOylation in Plant Defense. **(A)** SUMO conjugation/deconjugation cycle: SUMO is synthesized as a precursor that is processed at its C-terminal tail by the specific ULP proteases, releasing a SUMO mature form with a Gly–Gly motif at its C-terminus. Subsequently, SUMO is activated by the heterodimeric E1 activating enzyme, SAE1/SAE2, transferred to the E2 conjugating enzyme, and, finally, attached to a target lysine in the substrate. The target lysine is usually located within the consensus site ΨKxE/D (Ψ is a large hydrophobic amino acid, and x, any amino acid). This final step is facilitated by E3 ligase enzymes that interact with both SUMO-charged E2 and the substrate. SUMOylation is a reversible modification, and the same class of cysteine proteases involved in the maturation step catalyze SUMO excision from the substrate. Identified pathogen effectors establish distinct interactions with the plant SUMO machinery components. The viral proteins Rep and Nlb interact with the E2 conjugating enzyme. In addition, Nlb is also modified by SUMO3. The most abundant are bacterial effectors that display SUMO protease activity (AvrX4, XopD, NopD, HopBL1, and HopBL2). And the non-covalent interactions between the extracellular fungal MAMP EIX and tomato SUMO (the asterisk denotes that is an extracellular effector). **(B)** List of plant defense proteins shown to be regulated by SUMOylation. The molecular role of SUMO is indicated as SUMO conjugate, SUMO interactor or protein levels modulation by an indirect mechanism, such as regulation of mRNA levels.

Pathogens have evolved elaborate strategies to manipulate plant cells. Increasing evidence suggests that pathogens modify SUMOylation homeostasis of the host cells modulates innate immunity and promote virulence. Since SUMOylation is a multi-step signaling cascade, it provides an opportunity for the pathogen effectors to inhibit SUMOylation at different stages (Figure 1).

### Plant SUMO Depletion by Fungal Effectors

Phytopathogenic fungi constitute a versatile group of eukaryotes that successfully colonize and cause devastating diseases in crops species. Unlike bacteria and viruses, which invade through natural openings or wounds, fungal pathogens release a huge number of effector proteins with enzymatic activities into the host’s extracellular environment. Genome-wide analyses suggest that, in comparison to all animal and plant pathogens, phytopathogenic necrotrophs have the largest secretomes, and biotrophs release the smallest secretomes upon infection in crop plants (Kim et al., 2016). The fungal secretome is predicted to carry out various enzymatic activities aiming to collapse the host cell wall and to develop self-defense and acquiring nutrients (Lo Presti et al., 2015). However, several secreted fungal effectors proteins are categorized as unknown due to the absence of any recognizable domain in their sequence (Rafiqi et al., 2012; Vivek-Ananth et al., 2018). This group of unknown effectors is less studied and their biological function is not yet known, but they are thought to play a crucial role in the fungal colonization of plant tissue. In response to the biotrophic attack, plants initiate a HR to contain the infection by accumulating ROS and promoting cell death at the infection site. In contrast, in response to necrotrophs, plants activate the ROS scavenging system to eliminate ROS excess stimulated by fungi to prevent cell death (Mengiste, 2012).

The first report on plant SUMOylation uncovered the protein-protein interactions between the tomato SUMO (T-SUMO) and the fungal effector ethylene-inducing xylanase (EIX; Hanania et al., 1999). EIX is secreted by the fungus *Trichoderma viride* and, upon recognition by tomato and tobacco sensitive strains, EIX elicits plant defense responses leading to programmed cell death. This defense response is achieved through the induction of ethylene biosynthesis, accumulation of pathogenesis-related (PR) proteins, phytoalexins, and membrane electrolyte leakage (Bailey et al., 1990). EIX was shown to interact with T-SUMO in yeast two-hybrid and *in vitro* pull-down experiments (Hanania et al., 1999), although the molecular consequences of this interaction are unknown. *In vivo*, EIX treatments caused the reduction of T-SUMO mRNA levels in tomato leaves. Conversely, overexpression of T-SUMO attenuated induction of ethylene biosynthesis and cell death in transgenic tobacco plants (Hanania et al., 1999). The biological significance of direct interactions between EIX and T-SUMO, as well as the cellular events that allow molecular contacts between an extracellular fungal effector, EIX, and an intracellular plant protein, T-SUMO, still need to be identified. Nevertheless, these studies provided the first evidence supporting a central role of SUMOylation in plant immunity.

Similarly, in infection experiments of *Arabidopsis* by the necrotrophic fungi *Plectosphaerella cucumerina*, a depletion of free SUMO and SUMO conjugates were observed in the first 48 h after plant inoculation with fungal spores. It has been suggested that this depletion is the result of an unknown post-transcriptional mechanism that also affected the E1-large subunit SAE2 and the SCE1 conjugating enzymes turnover (Castaño-Miquel et al., 2017). Although future studies are needed to uncover this mechanism, these results contribute to delineate a model of a fungal infection that involves the degradation of SUMO from the host as a mechanism of pathogenicity.

### Bacterial Effectors Displaying SUMO Protease Activity

The secretion of effectors into the host cells, using the type-III secretion system (TTSS), is a crucial component of bacterial infection strategy. Among the effectors, cysteine proteases are present in several phytopathogenic bacteria such as *Xanthomonas campestris*, *Pseudomonas syringae*, or *Ralstonia solanacearum* (Hotson and Mudgett, 2004), which are discussed in the following subsections.

*Xanthomonas campestris* pathovar *vesicatoria* (*Xcv*) is the causal agent of bacterial spot disease in tomato (*Solanum lycopersicum*) and pepper (*Capsicum annum*) plants. During infection, *Xcv* delivers the SUMO proteases AvrXv4 and XopD into the plant cells (Hotson et al., 2003). AvrXv4 belongs to the C55 family of cysteine protease that is exemplified by the YopJ peptidases. Initial observations determined that the *Yersinia pestis* effector YopJ possessed SUMO protease activity (Orth et al., 2000), but subsequent studies demonstrated that YopJ acts as an acetyltransferase (Mukherjee et al., 2006) that affects the protein secretion pathway (Bartetzko et al., 2009). It is remarkable that the SUMO isopeptidase activity of AvrXv4 has only been established *in planta* and, not *in vitro* (Roden et al., 2004). Based on these findings, AvrXv4 is speculated to be the only member of YopJ-like effectors with SUMO protease activity, and it is unclear whether it also possesses acetyltransferase activity. The other effector, XopD, is a modular protein containing an N-terminal DNA-binding domain, two ethylene-responsive element-binding factor-associated amphiphilic repression (EAR) transcriptional repressor motifs and a C-terminal SUMO protease domain. XopD belongs to the C48 family of cysteine proteases, in the same way as the bona fide ULP SUMO proteases (Hotson et al., 2003).

After translocation, AvrXv4 localizes to the cytosol while XopD is present mainly at nuclear foci (Hotson et al., 2003; Roden et al., 2004), but both effectors induce depletion of SUMO conjugates in transient expression experiments to the same extent (Roden et al., 2004). Considering that endogenous SUMO conjugates are enriched in the nucleus, AvrXv4 should translocate to the nucleus or stimulate SUMO deconjugation through an indirect mechanism, although there is no data to support either option. However, XopD localizes to the nucleus, the same subcellular compartment as most of the plant SUMO conjugates (Más et al., 2020). These observations, together with the failure to reconstitute AvrXv4 SUMO protease activity *in vitro*, supports the hypothesis that AvrXv4 and XopD have distinct modes of action. In contrast to other ULP orthologs, XopD displays high substrate specificity and only display peptidase and isopeptidase activity on plant SUMO isoforms (Hotson et al., 2003; Chosed et al., 2007). Additional analyses of the XopD N-terminal regulatory domain revealed the presence of a DNA binding domain (DBD) that mediates the repression of gene expression induced by SA and JA. Both XopD SUMO protease and DNA binding capabilities are necessary for delaying the pathogen-induced cell death and promoting *Xcv* growth (Kim J.-G. et al., 2008), highlighting the role of XopD as a modular protein (Kim et al., 2011). The relevance of the distinct functional domains in virulence is supported by studies on the *Xanthomonas campestris* pv. *campestris* (*Xcc*) *8004* XopD ortholog, which does not contain the N-terminal domain. Heterologous expression of XopD*_*Xcc*__8004_* in *Arabidopsis* resulted in SA-dependent defense response elicitation and *Xcc8004* growth suppression. In accordance with its negative role in pathogenesis, XopD-deficient *Xcc8004* proliferated more actively than wild type *Xcc8004* during *Arabidopsis* infection. The absence of the N-terminal domain in *Xcc8004* not only compromised its virulence but elicited plant immunity (Tan et al., 2015).

In addition to repression of SA signaling, the bifunctional *Xcv* XopD also represses ethylene production upon infection acting as a “tolerance factor” during infection. XopD represses ethylene production by inhibiting the expression of the tomato ethylene biosynthetic genes *ACO1*, *ACO2*, and *ACS2* (Kim J. G. et al., 2013). Consistently, *Xcv* ΔxopD elicits ethylene production, by upregulation of ethylene biosynthetic genes, resulting in reduced bacterial proliferation. Degradation of ethylene by heterologous expression of bacterial ACC deaminase gene limits symptom development and restores *Xcv* Δ*xopD* growth. Mutagenesis analyses revealed that the XopD N-terminal non-specific DBD, the C-terminal SUMO protease domain and the central EAR motif are required to suppress ethylene production upon infection, although the SUMO protease is quantitatively less important (Kim J. G. et al., 2013).

Further investigations identified SlERF4 as a SUMO substrate targeted by XopD (Kim J. G. et al., 2013). The SlERF4 tomato transcription factor, which belongs to the APETALA2/Ethylene-Responsive Factor (AP2/ERF) family (Licausi et al., 2013), is induced by ethylene and upon *Xcv* infection. SlERF4 is SUMOylated at a single site and SUMO removal by XopD results in SlERF4 destabilization. As consequence, SlERF4-mediated ethylene production was impaired upon infection with *Xcv*, while infection with XopD deficient-*Xcv* resulted in increased plant ethylene production and bacterial growth inhibition (Kim J. G. et al., 2013). In this pathosystem, XopD represses SIERF4-dependent transcriptional induction and elicitation of defense responses.

Additional studies addressing the role of XopD from *Xcc* 8004 in *Arabidopsis* identified HFR1 as a XopD target. HFR1 is a basic helix-loop-helix transcription factor involved in photomorphogenesis (Tan et al., 2015). HFR1 and XopD co-localize in nuclear speckles and monoSUMOylation of HFR1 is reversed by XopD *in vitro*. Although these studies revealed a novel role of HFR1 in plant immunity, the molecular and biological consequences of HFR1 SUMOylation and regulation by XopD*_*Xcc*__8004_* remain unknown (Tan et al., 2015). In addition, the basic helix-loop-helix (bHLH) transcription factor bHLH132 is responsive to the XopD effector secreted by *X. euvesicatoria* in tomato (Kim and Mudgett, 2019). The expression of bHLH132 is highly induced by XopD SUMO protease activity and is required and sufficient for the resistance against *X. euvesicatoria* infection (Kim and Mudgett, 2019).

The presence of the XopD family effectors has also been identified in pathogens isolated from woody hosts. *Pseudomonas savastanoi* pv. *savastanoi* NCPPB 3335 is the causal agent of the olive knot disease that is characterized by the death of branches and progressive weakening, resulting in estimated production losses of 1.3% in Spain (Quesada et al., 2012). Two XopD-like proteins, HopBL1 and HopBL2, are secreted through the type III secretion system. Among them, HopBL1 is the closest homolog to XopD. Similar to XopD, both HopBL1 and HopBL2 harbor a C-terminal XopD-like SUMO protease domain (Kim et al., 2011). Their characterization in heterologous systems showed that HopBL1 and HopBL2 are likely involved in pathogen perception and interfere with defense responses exemplified by a reduction of ROS production and callose deposition. Their conservation suggests that both effectors play a relevant role in bacterial interactions with plant woody species (Matas et al., 2014).

Besides virulent bacteria, functional TTSS has also been identified in bacteria that establish a symbiotic relationship with legumes resulting in the formation of root nodules. These bacteria, known as rhizobia, fix and delivers atmospheric nitrogen to the host plant in exchange for carbon assimilates and nutrients (Udvardi and Poole, 2013). Nodulation outer proteins, Nop, are rhizobial T3 effectors responsible for the establishment and maintenance of the symbiosis. Homology analyses identified NopD from *Bradyrhizobium* sp. *XS1150* as a XopD ortholog. NopD has also specificity for plant SUMO, except for the most divergent *Arabidopsis* isoforms SUMO3 and SUMO5. Like XopD, NopD transient expression induces necrosis in tobacco leaves and localizes to nuclear foci. Nodulation experiments showed that NopD SUMO protease activity behaves as an asymbiotic factor and, *Bradyrhizobium* sp. *XS1150* carrying mutations in NopD locus induced an increment in the number of nodules and the nodule biomass per plant (Xiang et al., 2020).

### Viruses Hijack Plant DNA Replication Machinery

Viruses are among the simplest pathogens found in nature. They are obligate intracellular parasites unable to generate energy or to replicate outside the cell of a host. They are usually classified into six different groups based on the architecture of their genome. These groups include reverse transcribing viruses, double-stranded RNA (dsRNA), negative-sense single-stranded RNA (ssRNA-), positive-sense single-stranded RNA (ssRNA+), single-stranded DNA (ssDNA), and double-stranded-DNA (dsDNA). Plant viruses have representatives in all these groups being the ssRNA+ the most abundant. Despite their distinct mechanism of infection and replication, all viruses contain reduced genomes coding for a limited number of proteins. As a consequence, viruses require the manipulation of the host machinery to replicate and complete their life cycle and to prevent or counterattack the plant defense mechanisms. To do so under those space constraints, viruses encode multifunctional proteins (Trinks et al., 2005).

By targeting PTMs, viruses can potentially remodel pre-existing proteins and change their function, activity, or subcellular localization, modify the composition of protein complexes or, generate or disrupt previous protein-protein interactions. The plant PTM machinery have also evolved various counter mechanisms to subdue incoming viral proteins (Ribet and Cossart, 2010). For example, host-mediated phosphorylation of the *Turnip yellow mosaic virus* and *Tomato yellow leaf curl virus* proteins target them for degradation (Jakubiec et al., 2006; Shen et al., 2011).

As cell invaders, viruses have evolved mechanisms to modulate the conserved host cell SUMOylation system for successful infection. Most of our current knowledge about the mechanisms by which viruses exploit the SUMO machinery during the host-pathogen interaction is based on data from human/mammalian cells (Lowrey et al., 2017; Wilson, 2017). The identified molecular mechanisms involve the SUMOylation of viral proteins, as well as the alteration of the host SUMOylation to modify the normal growth, development, and defense mechanisms of the host. In plants, the role of SUMOylation in viral infection is less known, and most of the reported studies have focused on the *Geminiviridae* and *Potyviridae* virus families.

Geminiviruses are a large family of circular ssDNA viruses infecting a broad range of plants including many relevant crops (Rojas et al., 2005). This family of viruses is classified into nine different genera, being the Begomovirus genus the largest group (comprising > 320 virus species). The genome of these viruses usually encodes for 5–8 proteins through the generation of several viral transcripts under the control of promoters generally located within the intergenic region, however, none of these has a DNA polymerase activity. Hence, they replicate in infected plant cells nuclei using the host’s DNA polymerase. All Begomoviruses encode for the Rep multifunctional protein, also known as AL1, AC1, or C1. Rep is essential for their replication, mediates the recognition of its cognate origin of replication, is required for initiation and termination of viral DNA synthesis, and acts as a DNA helicase (Hanley-Bowdoin et al., 1999). Rep has been described to induce the accumulation of the host replication machinery, but also interacts with many host proteins, such as the cell cycle regulator retinoblastoma-related protein (RBR; Xie et al., 1995), the DNA replication protein PCNA (proliferating cellular nuclear antigen), or histone H3 (Kong and Hanley-Bowdoin, 2002).

The evidence of SUMOylation in plant viral infection was first demonstrated in *Yellow leaf curl sardinia virus* (TYLCSV) in *N. benthamiana*. Using yeast two-hybrid assays, the SUMO E2 conjugating enzyme 1 (NbSCE1) was found to interact with the TYLCSV Rep protein. Similarly, Rep proteins from *Tomato golden mosaic virus* (TGMV) and *African cassava mosaic virus* (ACMV) were also identified and validated to be physically interacting with NbSCE1 (Castillo et al., 2004). The specific interaction region was mapped to the N-terminal half of Rep, extending from residues 56 to 114 that defines a core region (amino acids 56–85) and a support region (amino acids 85–114; Sanchez-Duran et al., 2011). Furthermore, analysis of single and double mutants of the two lysines located in this region (K68 and K96) showed a critical role for the interaction with SCE1, both in yeast two-hybrid assays and *in planta*. Interestingly, these mutations had no impeding effect on other protein functions like oligomerization, DNA binding, or DNA cleavage, and did not affect interaction with other viral proteins such as REn (or AL3), or host proteins such as RBR (Sanchez-Duran et al., 2011). Further analysis of these lysine residues revealed that they play a key role in the nuclear localization of Rep proteins in some viruses, such as TGMV (Maio et al., 2019). The lysine mutation reduces viral DNA accumulation and virus infectivity in plants (Sanchez-Duran et al., 2011; Maio et al., 2019), suggesting that the Rep-SCE1 interaction could be required for viral DNA replication. The same negative effect on virus replication, but not on viral movement, is observed in plants with altered levels of SUMO (both reduced by silencing or overexpressed; Castillo et al., 2004), suggesting a key role of SUMOylation in viral replication and hence infectivity.

In mammalian DNA viruses, subnuclear localization of early viral protein complexes seemingly depends on SUMOylation. In contrast, geminivirus Rep is not modified by SUMO despite containing three putative SUMOylation sites on its sequence and being competent to interact with the SUMO E2 conjugating enzyme (Sanchez-Duran et al., 2011; Arroyo-Mateos et al., 2018). Instead, Rep is able to reduce the SUMOylation state of the plant PCNA, a cofactor that orchestrates genome duplication and was previously described to be up-regulated in the presence of Rep (Egelkrout et al., 2001). In reconstituted SUMOylation assay in *Escherichia coli* and in plants, SlPCNA was SUMOylated at lysines K164 and K254. In the presence of Rep, SUMOylation repression is only observed for SlPCNA, suggesting that this is a specific alteration and that Rep-SCE1 interactions do not alter global SUMOylation in plants (Arroyo-Mateos et al., 2018). Moreover, Rep mutants that are unable to interact with SCE1 still inhibit the SUMOylation of PCNA. Based on the previous information available from other systems like yeast, it has been suggested that the interaction between Rep and PCNA is the mechanism that prevents PCNA SUMOylation. In the absence of SUMOylation, PCNA loses its ability to recruit the DNA helicase Srs2, leading to higher levels of homologous recombination (HR). In consequence, an increase in viral recombination and replication efficiency could be favored (Arroyo-Mateos et al., 2018).

Following the initial studies conducted with geminiviruses, the role of SUMO modifications has been further explored in potyvirus, responsible for causing significant losses in a variety of crops. Potyvirus is single-stranded RNA virus that replicate in the cytoplasm of the host cell and usually encode 11 mature proteins in their genome (Valli et al., 2021). Some of the important potyviruses include *Turnip mosaic virus* (TuMV), *Tobacco etch virus* (TEV), and *Soybean mosaic virus* (SMV). A yeast two-hybrid screening of SCE1 from *Arabidopsis* against the 11 proteins codified in the genome of TuMV revealed a single interaction involving SCE1 and the viral Nlb (the nuclear inclusion b) protein, a RNA-dependent RNA polymerase (RdRp; Cheng et al., 2017). The SCE1-interacting domain mapped between residues 170–300 and represents a highly conserved region among potyviral NIbs. Later, similar interactions of SCE1 were also observed with Nlb proteins from TEV and SMV in *N. benthamiana*, highlighting a common role of SCE1 as a host factor essential for virus infection (Xiong and Wang, 2013). Viral Nlb locates in the nucleus of the host cell and contains a negatively charged amino acid-dependent SUMOylation motif (Yang et al., 2006), at least three potential SUMOylation sites (K148, K172, and K409), and two SUMO interacting motifs (SIM). In addition, SUMOylation by SUMO3 (but not SUMO1, SUMO2, or SUMO5) was detected using a reconstituted SUMO pathway in *E. coli* assays, probably at multiple positions (Cheng et al., 2017). SUMO3 is induced upon TuMV infection and mediates Nlb SUMOylation and its relocation from the nucleus back to the cytoplasm. Any alteration in SUMO3 levels suppress TuMV replication and mitigate viral symptoms. Nlb retargeting to the cytoplasm mediated by SUMOylation is essential for viral replication and suppression of host NPR1- mediated immunity (Xiong and Wang, 2013; Cheng et al., 2017). On the other hand, decreased levels of SCE1 in *Arabidopsis* plants inhibit the infection of TuMV and TRV (Xiong and Wang, 2013). Milder virus symptoms are also observed in SUMO3-defective plants (Cheng et al., 2017). Interestingly, transgenic *Arabidopsis* plants overexpressing SUMO3 also show fewer infection symptoms (Cheng et al., 2017), suggesting a dual role of SUMO3 in allowing viral infection (as a host factor) and activating defense mechanism. These complex interactions represent an example of how the viruses hijack and alter the host SUMOylation machinery to modify its own proteins, but also to suppress plant pathogen responses. Overall, there is strong evidence supporting that the viral pathogens seize the host SUMO machinery as virulence factors in plants to promote infection and proliferation. However, the mechanisms and components involved are still poorly understood.

## Plant SUMOylation to Counteract the Attack of Pathogens

Genetic studies using plants with altered levels of SUMOylation have consolidated SUMOylation as a key player in plant immunity. Recently, the identification of defense signaling components as SUMO targets has shed light on the molecular mechanisms involved in the role of SUMOylation in plant defense responses against bacteria, fungi, and viruses.

### SUMOylation Dynamics During Fungal Infection

Efforts to elucidate the role of SUMOylation in plant-fungal interactions have generated controversial results. Initial studies suggested that impairment of SUMOylation did not affect susceptibility to the necrotroph *Botrytis cinerea*. According to lesion development in infection experiments, *Arabidopsis siz1-3* plants displayed similar sensitivity to wild-type plants (Lee et al., 2007a). In a later study, the role of SUMOylation in response to fungal infections was studied in plants engineered to have different degrees of SUMOylation impairment. These plants expressed the SUMO E1 SAE2^UFDCt^ domain as a competitive inhibitor of the SUMO E1-E2 interactions, resulting in the inhibition of SUMOylation *in planta* (Castaño-Miquel et al., 2017). Fungal spore inoculation assays on intact plants showed that the SAE2^UFDCt^ expressing lines displayed higher susceptibility to *B. cinerea* and *P. cucumerina* infection in a dose-dependent manner, leading to increased plant death. Under the same infection conditions, *siz1-3* plants also exhibited enhanced sensitivity to necrotrophic fungal attack (Castaño-Miquel et al., 2017). Although controversial with the initial suggestion of a SIZ1-independent regulation of defense responses to fungal attacks, these results showing *siz1-3* susceptibility to necrotrophic pathogens are consistent with *siz1-3* characteristic elevated SA levels (Lee et al., 2007b).

Insights of SUMOylation in defense have also arisen from studies in crop species. Bean (*Phaseolus vulgaris*) productivity is compromised by the fungus causing anthracnose (*Colletotrichum lindemuthianum*). During the characterization of compatible (plant susceptibility) and incompatible (plant resistance) interactions, SUMO was identified as an early expressed gene during incompatible interactions. SUMO mRNA levels increased in response to the infection of both compatible and incompatible during the first 6h post-inoculation, but with clear differences in the amplitude of the response, 1.7-fold in compatible versus 4.2-fold in incompatible interactions (Fraire-Velázquez et al., 2003). Considering that early events may be determinant for bean performance, SUMOylation could account for a key factor in the final outcome.

The wheat gene *TaS3* (*Triticum aestivum* susceptibility) was identified as a susceptibility gene to the powdery mildew fungus *B. graminis*. *TaS3* is an ortholog of the SUMO protease *ELS1* and was found highly expressed in the susceptible wheat cultivar Yumai 13, suggesting that increased SUMO deconjugation confers susceptibility. On the contrary, *TaS3* is expressed at very low levels in the resistant wheat cultivars Hongyou and Chiyacaos (Li et al., 2013). In addition, TaS3 expression was transiently induced after 6–24 h inoculation in the susceptible cultivar, highlighting its relevance during early infection. Consistently, *TaS3* suppression by RNAi resulted in decreased fungal penetration and increased plant resistance, establishing a positive correlation between wheat SUMOylation and fungal resistance (Li et al., 2013).

Upregulation of genes of the SUMOylation machinery pathway has also been identified in *Solanum tuberosum*–*Phytophthora infestans* interactions. This connection was established in comparative studies between *S. tuberosum* variants sensitive and resistant to *P. infestans*. The resistant variant studied, “Desiree/RB,” shared the same genetic background as the sensitive variant, Desiree, except for the presence of the resistance gene *Rpi-blb1* derived from the wild species *Solanum bulbocastanum*. In the resistant variant Desiree/RB, the SUMO conjugating enzyme *SCE1* was up-regulated while the SUMO protease *ESD4* was down-regulated. These results suggest that “Desiree/RB” resistance to infection by *P. infestans* depends, at least in part, on its ability to initiate a rapid generalized increase of SUMO conjugates by increasing SUMOylation (*SCE1* upregulation) and decreasing deconjugation (*ESD4* downregulation) in response to infection (Colignon et al., 2017).

The central role of SUMOylation in defense against fungal colonization was further confirmed in a study showing the activation of the soybean SUMOylation pathway in response to another soil-borne oomycete, *Phytophthora sojae*. Soybean GmSUMO2/3, GmSAE1b, and SUMO conjugates accumulation was observed specifically in the roots of the resistant soybean varieties exposed to *P. sojae* infection. These results also support a positive association of SUMOylation in conferring enhanced resistance against the *P. sojae* (Li et al., 2019).

Similar associations have been established in genomic studies aiming to identify genes in Danish ash trees that confer resistance to the invasive fungus *Hymenoscyphus fraxineus*. The disease caused by this fungus is known as ash dieback and it has a major impact on the reduction of European ash trees. The identification of the SUMO conjugating enzyme, SCE1, in close proximity to SNPs associated with ash tree resistance to ash dieback suggested a positive role of SUMOylation in defense against *H. fraxineus* (Stocks et al., 2019).

Additional evidence supporting a positive role of SUMOylation in fungal resistance arose from metabolomics. The study of molecules accumulated in wheat and barley variants showing some level of resistance to *Fusarium graminearum* resulted in the identification of resveratrol, a candidate inhibitor of SUMO proteases (Surendra and Cuperlovic-Culf, 2017). Whether the mechanism of action of resveratrol targets plant or fungal SUMO proteases, or both, require further investigations.

Overall, these studies in model and crop species emphasize the importance of SUMOylation on necrotrophic fungal resistance and suggest that increased SUMOylation confers higher protection.

#### SUMOylation in Defense Responses Elicited by the *Trichoderma viride* Effector EIX

The EIX fungal effector is recognized by tomato plant cells through the membrane receptor LeEIX2, which belongs to the family of leucine-rich-repeat-like-protein. Upon EIX binding, LeEIX2 needs to enter the endocytic pathway in order to elicit the HR (Ron and Avni, 2004). Several studies using heterologous proteins showed that endocytosis of the LeEIX2-EIX complex is inhibited by EHD2, an *Arabidopsis* EH domain- containing protein. Initially, it was found that EHD2 binds the cytoplasmic domain of the LeEix2 receptor and inhibits its internalization and signaling (Bar and Avni, 2009). Later, a potential SUMOylation motif was identified in EHD2 and, shown to be required for inhibiting LeEIX2-EIX endocytosis and subsequent defense responses, including ethylene production, in tobacco transient expression experiments (Bar et al., 2014).

The synthesis of the ethylene precursor 1-aminocyclopropane-1-carboxylic acid, ACC, is a regulatory step catalyzed by the family of ACC synthases (ACS). In tomato, the fungal elicitor EIX causes an increase in the activity of LeACS2, leading to ethylene production (Matarasso et al., 2005). Molecular analysis aiming to identify transcriptional activators of *LeACS2* resulted in the identification of a cytosolic cysteine protease, LeCp, which belongs to the family of vacuolar processing enzymes (VPE). VPEs perform multiple cellular functions ranging from maturation of seed storage proteins to induction of PCD as part of developmental programs or defense under biotic and abiotic stress (Hatsugai et al., 2015). Obtained results demonstrated that LeCp functions as a transcriptional activator necessary for LeACS2 upregulation in response to EIX. As LeCp does not contain any nuclear localization signal that would facilitate its transcriptional activator role, the authors hypothesized that SUMO could mediate its nuclear translocation. Accordingly, tomato T-SUMO was shown to interact with LeCp in yeast-two hybrid assays and, the disruption of a potential SUMOylation site in LeCp impaired its capacity to induce *LeACS2* transcription (Matarasso et al., 2005). Nonetheless, direct evidence of SUMOylation of LeCP remains to be determined.

Although SUMOylation to LeCp and EHD2 needs further validation, these results would suggest that SUMO could have opposing roles in repressing (Hanania et al., 1999; Bar et al., 2014) or promoting (Matarasso et al., 2005) ethylene production upon EIX elicitation in tobacco leaves.

#### SUMOylation in JA Mediated Defense Against Necrotrophs

Jasmonate signaling relies on proteasome degradation of transcriptional repressor JAZ proteins mediated by the F-box protein CORONATINE INSENSITIVE 1 (COI1). COI1-JAZ complex function as co-receptor of the bioactive jasmonate JA-Ile, which acts as a molecular glue to facilitate JAZ degradation (Sheard et al., 2010). In general, the SA and JA signaling mechanisms are considered to have opposing functions (Glazebrook, 2005). As previously mentioned, SA activates defense against biotrophic pathogens, whereas JA is effective against necrotrophic and herbivorous insects (Glazebrook, 2005; Howe and Jander, 2008). The enhanced resistance of the mutated plants in OTS (overly tolerant to salt) SUMO proteases 1 and 2 (*ots1 ots2*) to biotrophic pathogens due to elevated SA (Bailey et al., 2016) led to the speculation that they might exhibit sensitivity to necrotrophs, whose invasion is limited by JA signaling pathway. Under basal conditions, *ots1 ots2* plants do not accumulate SUMO conjugates to the same extent as the SUMO protease *esd4-1* mutant plants (Murtas et al., 2003; Conti et al., 2008), suggesting that OTS1/OTS2 could have specialized functions *in vivo*. Supporting this hypothesis, initial reports showed that OTS1/OTS2 depletion confers hypersensitivity to salt stress (Conti et al., 2008). In *ex vivo* leaf assays, *ots1 ots2* plants showed susceptibility to the fungal pathogen *B. cinerea* (according to lesion diameter) and the arthropod herbivore spider mite (according to egg deposition), *Tetranychus urticae*. The opposite effect was not detected in OTS1-overexpressing lines, consistently with the early degradation of OTS1-HA observed at 6 h after *B. cinerea* infection.

JAZ repressors, exemplified by JAZ6, and COI1 appeared as potential molecular mediators of the regulation of JA signaling by SUMO (Srivastava et al., 2018). Under unchallenged conditions, constitutively expressed JAZ6 over accumulated in *ots1 ots2* mutant background and it was rapidly degraded reaching almost undetectable levels after 30 min of JA exposure. Interestingly, OTS1 levels were not affected by JA treatments. Assuming that OTS1 protease activity correlate with OTS1 protein levels, OTS1-mediated transition from JAZ6 SUMOylated to deSUMOylated states seems not to have a significant role in JA-induced degradation of JAZ6. On the contrary, in *ex vivo* experiments in response to *B. cinerea* infection, OTS1 was degraded and levels of free- and high molecular weight SUMO conjugated-JAZ6 increased (Srivastava et al., 2018). Given that *B. cinerea* induces JA production, it is intriguing the opposite effect that both treatments have on OTS1 and JAZ6 levels. A possible explanation could involve differences in endogenous active JA concentration present in both assays, although they were not determined. Also, infection experiments were performed in detached leaves, which is expected to induce JA biosynthesis as part of the wounding response (Wasternack, 2007) prior to fungal inoculation. Under these conditions, the opposite effect obtained in the levels of OTS1 and JAZ6 between the infection experiments and the JA treatments may be explained by possible desensitization of the cells to JA in the infection experiments. Supporting this hypothesis, it is described that cell-desensitization to JA occurs as a result of the negative regulatory loop involving JAZ and MYC2 proteins (Chico et al., 2008). Another hypothesis would suggest that OTS1-HA/JAZ6 molecular regulation under *B. cinerea* infection is independent of endogenous JA synthesis, raising the question of which mechanism would modulate this distinct JA-dependent/independent regulation. Interestingly, COI1 promoted larger infection lesions when overexpressed in *ots1 ots2* in comparison to wild-type plants, suggesting that accumulation of SUMO conjugates in *ots1 ots2* would attenuate the COI1 effect in immunity. The identification of a SIM in COI1 led to the speculation of the role of OTS1 and OTS2 as a positive regulator of JA signaling by disrupting non-productive ternary complexes, JAZ-SUMO-COI, that would result in the destabilization of JAZ repressors and induction of JA signaling.

Although the proposed mechanism adds another layer of regulation to the COI1-JAZ signaling module very relevant to fine-tune the physiological responses to JA signaling, further investigations would contribute to reinforce this model. One unresolved question is the fact that differences in lesion development between plants expressing the COI1^V553A^ mutant defective in SUMO interactions and the native COI1 was only observed in the *ots1 ots2* background and not Col-0, suggesting that the SIM motif may have a minor contribution to COI1 activity in wild type background. Also, future studies addressing the relevance of JAZ6 SUMOylation in JA-mediated root inhibition or *B. cinerea* infection assays, including gene expression studies, will be very valuable to elucidate the contribution of JAZ6 SUMOylation to the regulation of JA signaling.

**FIGURE 2:**
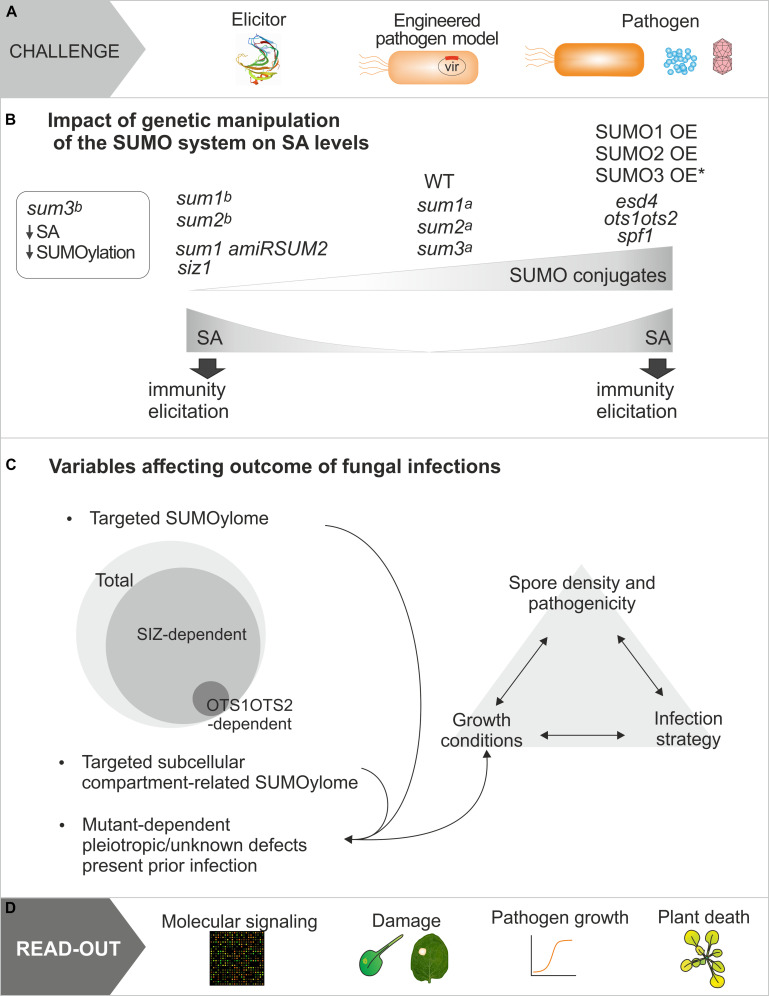
Multifactorial aspects affecting the study of the regulation of plant defense responses by SUMO conjugation. **(A)** Representation of approaches used to study plant defenses responses, which include molecule elicitation, pathogen model expressing a virulence effector from the pathogen of interest, or the pathogen of interest (bacteria, fungi, and viruses). **(B)** Different genetic manipulations leading to increase or decrease plant SUMO conjugation converge in salicylic acid accumulation and plant immunity elicitation. WT refers to levels in wild type plants. Mutated components of the SUMOylation machinery are indicated in italics. Single SUMO mutant plants (sum1, sum2, and sum3) have been reported not show [a, (van den Burg et al., 2010)], and to show [b, (Ingole et al., 2021b)] alterations in SA levels. **(C)** Summary of distinct experimental approaches used in studies analyzing the role of SUMOylation in defense against fungal pathogens and that may lead to the controversial conclusions generated. **(D)** Type of read-outs used in the studies discussed.

Overall, multiple factors account for the complexity of plant-fungal interactions. These involve the type of infection strategies, which may include a combination of initial biotrophic and late necrotrophic phases (Pétriacq et al., 2016); the experimental design (intact plant versus detached leaves, or elicitor versus pathogen of interest); the read-out approach to assess infection progression; the specific SUMOylome studied, and the dynamics of SUMOylation during infection and development. All these factors highlight the challenge to elucidate the molecular mechanisms that mediate the SUMO role in fungal defense (Figure 2).

### Alterations in SUMOylation Homeostasis Modify Defense Against Pathogenic Bacteria

During bacterial infection, two types of plant-pathogen interactions can be established: a compatible interaction (successful infection leading to disease), or an incompatible interaction (successful plant defense) that can trigger a HR mediated by effector recognition (Glazebrook, 2005). In compatible interactions, the pathogen is classified as virulent and the host plant, as susceptible. Most of the studies addressing the role of SUMOylation in plant defense against bacterial attack have used the strain *Pseudomonas syringae pv. tomato* DC3000 (*Pst* DC3000), which is virulent on *Arabidopsis thaliana* ecotype Col-0 (Whalen et al., 1991).

Initial studies determined that *Arabidopsis* plants harboring T-DNA insertions in the SUMO E3 ligase SIZ1 display constitutive systemic acquired resistance (SAR) characterized by elevated levels of SA, which also confers a dwarf phenotype (Lee et al., 2007b). Consistently, *siz1* mutant plants display increased resistance to *Pst* DC3000 (Lee et al., 2007b; Gou et al., 2017; Hammoudi et al., 2018). Similarly, *sum1-1* amiRSUM2 double mutant causes increased accumulation of SA, enhanced expression of PR genes and better resistance to *Pst* DC3000 (van den Burg et al., 2010). These results suggested that SUMOylation could be a negative regulator of SAR. However, plants engineered to accumulate SUMO conjugates by overexpression of SUMO1/2/3 (van den Burg et al., 2010), or loss of function in SUMO proteases, such as ESD4 (Villajuana-Bonequi et al., 2014) or OTS1/OTS2 (Bailey et al., 2016), also contain increased SA levels. In addition, the fact that independent studies generate opposite observations using the same mutant plants highlights the complexity of the system and, the impact of plant growth conditions on the results. A recent study has reported that mutations in SUMO1 confer a dwarf phenotype that correlates with SA accumulation (Ingole et al., 2021b), which was not previously observed by others (Saracco et al., 2007; van den Burg et al., 2010). In fact, SUMO1 and SUMO2 have been considered functional redundant isoforms based on their high protein sequence homology and the lack of phenotypic alterations in single *sum1* and *sum2* plants. These divergent results also affect plants with SUMO3 depletion. While *sum3* plants were initially reported not to have alterations in SA levels (van den Burg et al., 2010), recent results indicated that SUMO3 depletion results in a significant decrease in SA levels (Ingole et al., 2021b). Despite not knowing the factors responsible for this variability, the newly uncovered phenotypes allowed to study genetic interactions between *sum1* and *sum3* plants. The authors concluded that SUMO1/2 and SUMO3 play a negative and a positive role in immunity, respectively, (Ingole et al., 2021b). Consistently, loss of SUMO3 suppresses developmental defects and increased immunity of *sum1* mutant (Ingole et al., 2021b). On the other hand, SA treatments induce a reduction of recombinant OTS1 levels expressed in *Arabidopsis* and an accumulation of SUMO1/2 conjugates in Col-0. Also, the accumulation of SA in *ots1 ots2* plants correlates with increased resistance to *Pst* DC3000 (Bailey et al., 2016), suggesting a protective role of SUMOylation, opposite to the conclusions generated from the above-mentioned *siz1-3* studies. Consistent with this protecting role during *Pst* DC3000 infection, accumulation of SUMO1 conjugates correlate with upregulation of SUMOylation genes (*SAE2, SCE1*, and *SIZ1*) and downregulation of SUMO proteases genes (*ESD4* and *ELS1*; Ingole et al., 2021a). Overall, these results suggest that *in vivo* SUMOylation homeostasis must be under tight regulation in order to preserve cellular functions, and over or under accumulation of SUMO conjugates have deleterious effects leading to SA accumulation and, consequently, to a constitutive SAR (Figure 2). In addition to transcriptional regulation and protein turnover, SUMOylation homeostasis can be modulated by PTM on the SUMO machinery components. Supporting this hypothesis, a recent study showed that the SUMO conjugating enzyme SCE1 is modified by S-nitrosylation in response to *Pst* DC3000 infection. This modification inhibits SUMOylation and the authors postulate that it constitutes a molecular mechanism to release the repression of basal immunity mediated by SUMO (Skelly et al., 2019).

Beyond *Arabidopsis*, the role of SUMOylation in defense to bacterial infection has also been studied in tomato plants. *Clavibacter michiganensis* ssp. *michiganensis* (*Cmm*) is the causal agent of bacterial wilt and canker in tomato (*S. lycopersicum*). The analysis of *Cmm* resistant tomato varieties found an upregulation of SUMO E2-conjugating enzyme in *Solanum peruvianum* in response to infection by *Cmm* (Lara-Ávila et al., 2012). Induction of SCE1 silencing prior to *Cmm* infection conferred higher susceptibility to *S. peruvianum*, supporting a positive role of SUMOylation in plant immunity (Esparza-Araiza et al., 2015).

In the last years, different studies have focused on the characterization of specific regulators of defense responses as SUMO targets, as described below.

#### SUMO Associates With Pathogen Sensing Receptors

Another study has provided evidence that SUMO proteins can act in sync with the pathogen sensing receptors, such as Flagellin-Sensitive 2 (FLS2). FLS2 is a pattern recognition receptor consisting of three domains: the extracellular leucine-rich repeat (LRR) that primarily recognizes the bacterial flagellin (flg22), a transmembrane region, and the intracellular serine/threonine kinase domain. Flg22 recognition by FLS2 triggers FLS2 association with the LRR-RK BRI1-associated kinase 1 (BAK1), which acts as a co-receptor (Sun et al., 2013). The flg22-induced FLS2-BAK1 heterodimer initiates a series of *trans*-phosphorylation events that results in the FLS2- and BAK1- dependent phosphorylation of BOTRYTIS-INDUCED KINASE 1 (BIK1), a receptor-like cytoplasmic kinase, and the later dissociation of BIK1 from FLS2 to elicit plant immunity (Zhang et al., 2010). SUMO has been shown to modify the FLS2 kinase domain in response to flg22 recognition, contributing to BIK1 release from the FLS2-BAK1 complex. Plants expressing SUMOylation-deficient FLS2 variant are more susceptible to *Pst* DC3000, suggesting a SUMOylation requirement to initiate immune responses (Orosa et al., 2018). SUMO conjugate-FLS2 levels were regulated by the specific deSUMOylating enzyme Desi3a. During bacterial infection, flagellin elicited a rapid reduction in Desi3a levels causing the accumulation of the hyperSUMOylated FLS2, which promotes dissociation of BIK1 from the co-receptor complex to activate the intracellular immune signaling response (Orosa et al., 2018). This model, although it is still unknown the mechanism that transduces the signal to Desi3a and how it is degraded, provides a role of SUMO as a first responder in plant defense by regulating the pathogen recognition receptor FLS2.

#### Plant SUMO Regulates Defense Through the TPR1/SNC1 Signaling Module

SNC1 (Suppressor of *npr1-1*, constitutive 1) is a Toll interleukin-1 receptor nucleotide binding-Leu-rich repeat-type (TIR-NB-LRR-type) NLR protein and its overexpression causes constitutive activation of plant immune responses (Li et al., 2001; Stokes et al., 2002). The enhanced resistance to *Pst* DC3000 of *siz1-3* was found to be partially dependent on the SNC1 (Gou et al., 2017; Hammoudi et al., 2018). Overexpression of SIZ1 reduces SNC1 mRNA levels and SNC1 protein accumulation, partially rescuing dwarfism of the gain-of-function mutant *snc1-1* (Zhang et al., 2003; Gou et al., 2017). Subsequent demonstration of SNC1 SUMOylation in tobacco transient expression experiments, suggested that SNC1 degradation could be the molecular consequence of its SUMOylation (Gou et al., 2017). According to these results, the SUMO system would reduce SNC1 activity at the transcriptional and post-transcriptional level, resulting in the attenuation of SAR.

Relief of transcriptional repression to initiate signal transduction is a recurring theme in plant regulatory signaling pathways. In SNC1-mediated immunity, the transcriptional corepressor TOPLESS-RELATED 1 (TPR1) was identified as an SNC1-interacting protein. Previously, TOPLESS and TPR1 were also identified as putative SUMO substrate in an affinity purification screening analysis (Miller et al., 2010). TPR1, together with SNC1, activates the immune responses by repressing the expression of negative regulators such as DEFENSE NO DEATH 1 and 2 (DND1 and 2), which encode cyclic nucleotide-gated ion channels (Zhu et al., 2010; Chin et al., 2013). A recent study highlighted that SIZ1 mediated SUMOylation of TPR1 represses its activity under non-stress conditions. The SUMOylation of TPR1 at two lysine residues, K282 and K721, inhibits its interaction with HISTONE DEACTYLASE 19 (HDA19) and subdues its transcriptional corepressor activity, which results in the suppression of autoimmunity under non-pathogenic conditions (Niu et al., 2019).

#### Regulation of SA Perception by SUMO

NON-EXPRESSOR OF PR GENES 1 (NPR1) is a positive regulator of SAR that, together with other members of the NPR-like family, have emerged as a bona fide SA receptor. A recent review has addressed key molecular and biological aspects of NPR1 family (Backer et al., 2019). Upon activation, NPR1 is translocated to the nucleus and recruits TGA transcription factors to induce expression of PR genes, acting as a transcriptional cofactor (Berens et al., 2017). NPR1 also induces the expression of WRKY genes, and WRKY-regulated genes, some of which are involved in SAR attenuation by repressing SA-induced genes (Wang et al., 2006). The positive regulation of NPR1 by WRKY genes highlight the intricate network of positive and negative feedback regulatory loops that secure the duration and amplitude of defense responses. In the cytosol, NPR1 is competent to inhibit JA-dependent plant responses, highlighting the existence of specific roles of NPR1 depending on the subcellular compartment (Spoel et al., 2003). As a key player in plant immunity responses, NPR1 is subjected to multiple regulatory layers (Backer et al., 2019; Zavaliev et al., 2020), including modification by SUMO (Saleh et al., 2015).

NPR1 has been proposed to be regulated by SUMO through covalent modification and SIM-mediated interactions. NPR1 interacts specifically with SUMO3 isoform, known to be highly induced upon SA treatments (van den Burg et al., 2010), and is modified by SUMO3 in response to SA *in planta* (Saleh et al., 2015). NPR1-SUMO3 interactions are mediated by a SIM motif, SIM3, which is required for NPR1 SUMOylation in the *E. coli* reconstituted system, in yeast, and *in planta*. Unfortunately, the mutagenesis analysis failed to identify the lysine residues in NPR1 having a role as SUMO acceptors, preventing the study of a SUMOylation-deficient NPR1 variant. These results highlight the limitations of sequence prediction algorithms to identify SUMO acceptor lysine residues located at sites with low conservation of the SUMOylation consensus sequence. Instead, the authors focused on the study of the NPR1^sim3^ mutant variant that is not competent to interact with SUMO3 and fails to be SUMOylated *in planta*. The role of SIM motifs in SUMO targets as facilitators of SUMOylation is a well-established mechanism in the SUMO system (Knipscheer et al., 2008). In addition, distinct phosphorylation sites in NPR1 prevent, S55/59, or stimulate, S11/15, NPR1 SUMOylation, providing an amplification loop. One molecular consequence of SUMO regulation of NPR1 involves the modulation of protein-protein interactions. The presence of the SIM3 motif is required for efficient interaction with the SAR positive regulator TAG3, while SIM3 deletion results in specificity changes that favor interactions with the SAR negative regulator WRKY70. Further validation showed that SIM3 deletion also results in NPR1 accumulation in the cytosol and defense responses impairment. Although the proposed mechanism is very elegant, considering that endogenous NPR1 will constitutively contain a functional SIM3 motif, the physiological relevance of this SIM3-dependent switch would imply conformational changes that will be exposing or hiding the SIM3 motif to interacting partners. It cannot be ruled out, and maybe more plausible, that the SIM3-dependent NPR1 switch will depend on differential SUMO3 modification of NPR1 interacting partners. It is remarkable that NPR1^sim3^ mutant has a predominant cytoplasmic localization after SA treatment, as opposed to the mainly nuclear of native NPR1, suggesting an additional role of SUMO in NPR1 subcellular localization (Zavaliev et al., 2020). Although challenging, future structural studies will be crucial to understand the molecular implications of the multiple PTMs of NPR1. In particular, it will be interesting to uncover if NPR1-SUMO3 interactions and NPR1 SUMOylation occur intramolecular, intermolecular, or both, and their effect on the molecular determinants that mediate NPR1 interactions with other defense components. Nonetheless, these studies established a role of SUMO3 as an NPR1 interactor, distinct from the essential SUMO1/2 paralogs.

## Extended Overview of the SUMOylome in Plant Defense

**FIGURE 3:**
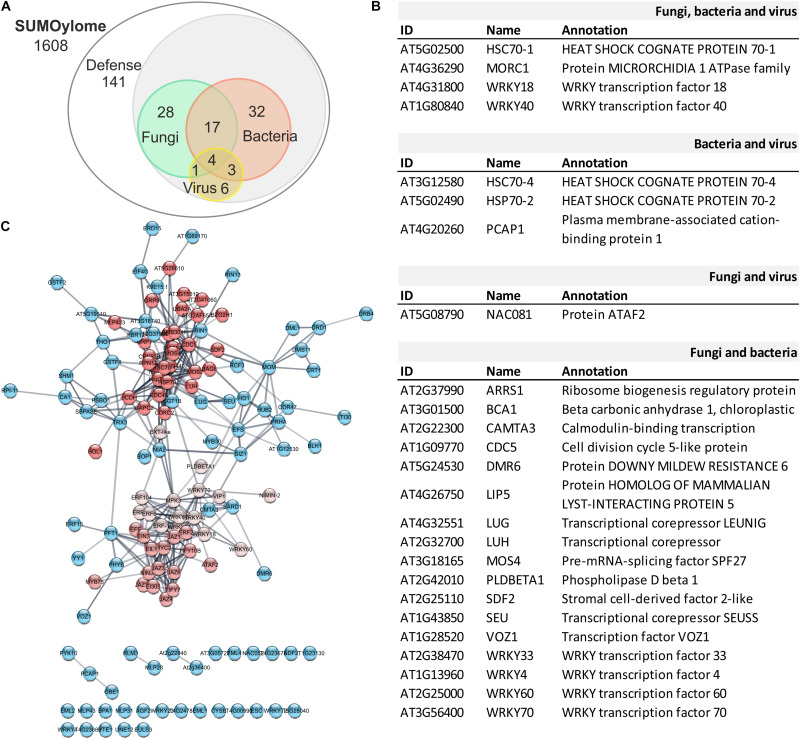
Network Analysis of the defense SUMOylome. **(A)** Venn diagram showing distribution of potential SUMO targets with a role in defense (141) responses to pathogenic fungi (28), bacteria (32), or virus (6). The proteins involve in defense responses against multiple pathogens are indicated at the intersections. **(B)** List of proteins included in the indicated intersections. **(C)** Interaction network analysis of the 141 proteins with a role in defense responses. The functional association obtained using the STRING web server was analyzed according to MCL cluster algorithm (Cytoscape). The three protein interaction clusters identified are indicated by different shades of red.

A comprehensive view of the scope of SUMOylation in plant-pathogen interactions can be achieved by defining the set of SUMOylated proteins in the cell (SUMOylome) within the confines of pathogen-specific cues. Recent non-targeted strategies enabled by technical innovations in mass spectrometry have delivered a large number of proteins that are candidates to be modified by SUMO. We have analyzed the SUMOylome in *Arabidopsis* to provide a resource of endogenous SUMO conjugates with a relevant role in plant defense and immunity. Taking advantage of publically available data, we constructed a database containing 1608 unique SUMO substrate candidates identified in proteomic approaches (Castro et al., 2016; Augustine and Vierstra, 2018; Rytz et al., 2018; Liu C. et al., 2019; Zhang et al., 2019; Qu et al., 2020; Zheng et al., 2020). Their role in plant immunity was established by performing a Gene Ontology term analysis. Among the identified 141 proteins with a role in defense, 32 are assigned exclusively to bacteria, 28 to fungi, and 6 to virus defense responses (Figure 3A). The intersecting group between bacteria and fungi consists of 17 proteins, 3 between bacteria and viruses, and 1 protein between virus and fungal defense.

Among the most versatile, 4 proteins were found to have a broad role in defense, independent on the pathogen and include, WRKY18, WRKY40, MORC1, and HSC70-1 (Figure 3B). The negative regulators of basal defense WRKY18 and WRKY40 belong to the large family of WRKY transcription factors known to have a role in PTI and ETI (Chi et al., 2013). WRKY18 and WRKY40 are close homologs sharing 60% of sequence identity and, together with WRKY60, form the WRKY IIa subclass. All three isoforms homodimerize and also interact with each other to form heterodimers, WRKY40/18, WRKY40/60, and WRKY18/60, which results in the modulation of their binding capacity to W-box sequences and biological role. Among them, WRKY40 displays the highest DNA binding activity that is enhanced by WRKY18, independently on the structural organization of the W-boxes analyzed. On the other hand, WRKY60 shows little DNA binding activity. When in heterocomplexes, WRKY60 diminishes WRKY40 binding activity and, depending on the W-boxes organization, enhances or reduces WRKY18 binding efficiency, providing a mechanism for target discrimination. A consequence of this interplay is that the enhanced resistance to *Pst* DC3000 conferred by WRKY18 overexpression is counteracted by WRKY40 or WRKY60 co-expression. On the other hand, constitutive expression of WRKY18 produced plants more susceptible to *B. cinerea*, and this susceptibility was enhanced by WRKY40 or WRKY60 co-expression (Xu et al., 2006), highlighting the complexity of the interplay between protein interacting partners and pathogen-dependent defense responses. Considering their role as potential SUMO targets, it is plausible that SUMOylation could contribute to modulate WRKY complexes formation to fine-tune defense responses.

Microrchidia (MORC) proteins have been described as epigenetic regulators and plant immune mediators in *Arabidopsis* (Dong et al., 2018). MORC proteins usually contain several distinct conserved domains features: an N-terminal catalytic ATPase module, composed of the conserved GHKL (Gyrase, Hsp90, Histidine kinase, MutL) and S5 fold domains, and a C-terminal domain containing one or more coiled-coil regions involved in protein-protein interactions (Dong et al., 2018). MORC1 interacts with immune components, including plant resistance proteins (R) and the PAMP recognition receptor FLS2. In addition, mutations in MORC1 leads to derepression of transposable elements and expression of silenced-genes (Moissiard et al., 2012), suggesting that MORCs enforce silencing by a mechanism independent of DNA methylation (Moissiard et al., 2012).

**FIGURE 4:**
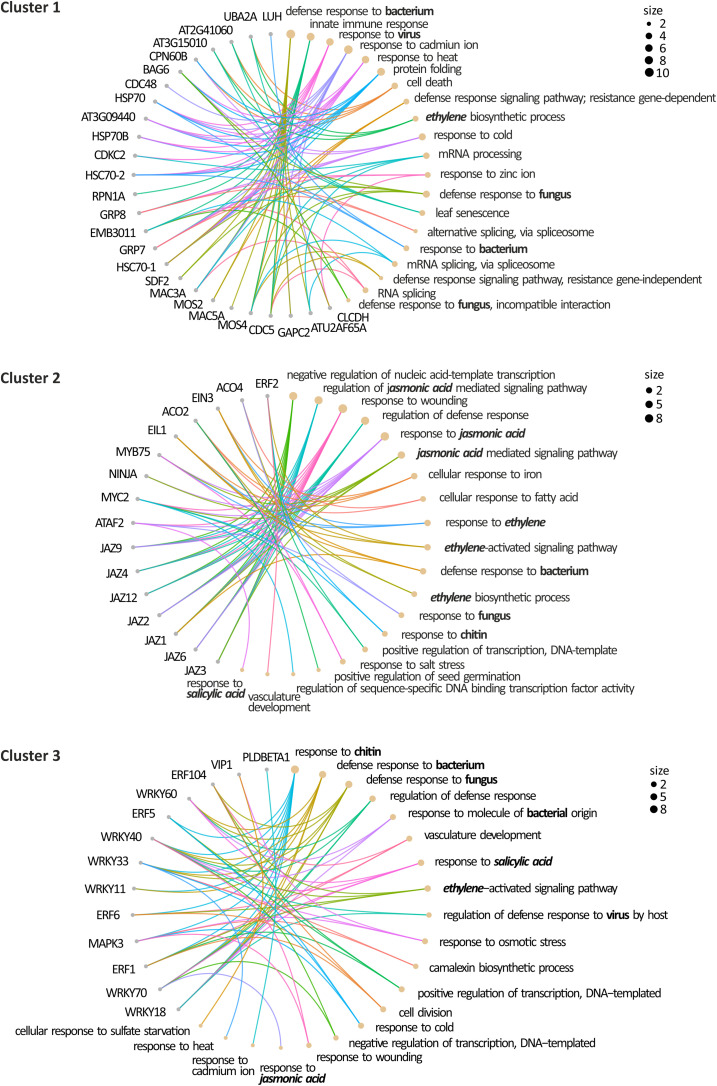
Gene Ontology Analysis of the defense SUMOylome. Gene ontology of the defense SUMOylome interactions: The proteins included in the clusters identified in Figure 3 were submitted to the DAVID Gene Ontology Enrichment Analysis tool (https://david.ncifcrf.gov/) using default parameters. The retrieve gene ontology terms are visualized on CirGO plots. The size represents the scale of the number of the analyzed genes that belong to the ontology. Functional categories related to defense hormones and responses to pathogens are in bold.

Folding and degradation of proteins are vital for the regulation of metabolic processes and stress responses. Correct folding and subsequent assembly into oligomers are necessary for functional enzymes and misfolded proteins can be refolded by chaperonins (Gou et al., 2015). Chaperones contribute to cellular proteostasis by regulation of *de novo* protein folding, assembly of macromolecular complexes, protein transport and degradation, and protein aggregate dissociation and refolding of stress-denatured proteins (Kim Y. E. et al., 2013). HSP70 is central to the cellular chaperone network, being the most abundant conserved molecular chaperones in all living organisms (Hartl et al., 2011; Kim Y. E. et al., 2013). In *Arabidopsis*, there are 18 genes encoding HSP70 proteins. The HSP70 family is divided into 2 subfamilies: DnaK and HSP110/SSE. The DnaK subfamily is formed by 13 members: 5 cytosolic HSP70 proteins, also called HSC70 (HSP70-1 to HSP70-5) and can be localized to the cytosol and nucleus; 3 are endoplasmic reticulum-localized luminal binding proteins, called BiPs (HSP70-11 to HSP70-13); 3 localize to plastids HSP70 (HSP70-6 to HSP70-8); and 2 are mitochondrial isoforms (HSP70-9 and HSP70-10). The HSP110/SS subfamily consists of 4 members (HSP70-14 to HSP70-17; Hartl et al., 2011; Kim Y. E. et al., 2013). All five members of the HSC70 group have been identified in proteomic studies as SUMO targets (Figure 4 and Table 2), with HSC70-1 showing a versatile role in defense responses to fungi, bacteria and viruses (Figure 3B). The role of the HSP70 family in plant defense has been determined considering their role as interactors of the immune receptor protein SNC1 (Gou et al., 2015), their capacity to disable resistance to virulent and avirulent pathogens when overexpressed (Noël et al., 2007), and their role as targets for pathogens effectors (Jelenska et al., 2007; Yang et al., 2017).

**TABLE 2:** List of gene contained in the interactome clusters.

ID	Gene	Annotation	Defense to
**CLUSTER1**			
AT4G36690	ATU2AF65A	U2 snRNP auxilliary factor, large subunit, splicing factor	Bacteria
AT2G46240	BAG6	A member of Arabidopsis BAG (Bcl-2-associated athanogene) proteins, plant homologs of mammalian regulators of apoptosis	Fungi
AT4G02640	BZO2H1	bZIP transcription factor family protein	
AT3G09840	CDC48	Encodes a cell division cycle protein, a member of AAA-type ATPases gene family	
AT1G09770	CDC5	Cell division cycle 5	Fungi, bacteria
AT5G64960	CDKC2	Cyclin dependent kinase group C2	Virus
AT1G65930	cICDH	Cytosolic NADP+-dependent isocitrate dehydrogenase	Bacteria
AT1G55490	CPN60B	Chaperonin 60 beta	Fungi
AT5G13010	EMB3011	RNA helicase family protein	Fungi
AT1G13440	GAPC2	Glyceraldehyde-3-phosphate dehydrogenase C2	Bacteria
AT2G21660	GRP7	Cold, circadian rhythm, and RNA binding 2	
AT4G39260	GRP8	Cold, circadian rhythm, and RNA binding 1	
AT2G43910	HOL1	HARMLESS TO OZONE LAYER 1	
AT5G02500	HSP70-1	Heat shock protein 70-1	Fungi, Bacteria, Virus
AT5G02490	HSP70-2	Heat shock protein 70-2	Bacteria, virus
AT3G09440	HSP70-3	Heat shock protein 70-3	Virus
AT3G12580	HSP70-4	Heat shock protein 70-4	Bacteria, virus
AT1G16030	HSP70-5	Heat shock protein 70-5	Virus
AT2G32700	LUH	LEUNIG homolog	Fungi, bacteria
AT1G04510	MAC3A	MOS4-associated complex 3A	Bacteria
AT1G07360	MAC5A	CCCH-type zinc fingerfamily protein with RNA-binding domain	Bacteria
AT1G24020	MLP423	Member of the Latex Protein family (MLP)-like protein 423	
AT1G33520	MOS2	D111/G-patch domain-containing protein	Bacteria
AT3G18165	MOS4	Encodes MOS4 (Modifier of snc1, 4)	Fungi, bacteria
AT2G20580	RPN1A	26S proteasome regulatory subunit S2 1A	
AT2G25110	SDF2	Stromal cell-derived factor 2-like protein precursor	Fungi, bacteria
AT3G56860	UBA2A	UBP1-associated protein 2A	
AT2G41060		RNA-binding (RRM/RBD/RNP motifs) family protein	
AT5G26610		D111/G-patch domain-containing protein	
AT3G15010		RNA-binding (RRM/RBD/RNP motifs) family protein	
**CLUSTER2**			
AT5G08790	ATAF2	NAC (No Apical Meristem) domain transcriptional regulator superfamily protein	Fungi, virus
AT1G05010	EFE	Ethylene-forming enzyme	Fungi
AT1G62380	EI305	ACC oxidase 2	
AT2G27050	EIL1	ETHYLENE-INSENSITIVE3-like 1	Bacteria
AT3G20770	EIN3	Ethylene insensitive 3 family protein	Bacteria
AT5G47220	ERF2	Encodes a member of the ERF (ethylene response factor)	Bacteria
AT1G19180	JAZ1	JAZ1 is a nuclear-localized protein involved in jasmonate signaling	Bacteria
AT5G20900	JAZ12	Jasmonate-zim-domain protein 12	
AT3G17860	JAZ3	Jasmonate-zim-domain protein 3	
AT1G48500	JAZ4	Jasmonate-zim-domain protein 4	
AT1G72450	JAZ6	Jasmonate-zim-domain protein 6	
AT1G56650	MYB75	Production of anthocyanin pigment 1	
AT1G32640	MYC2	Basic helix-loop-helix (bHLH) DNA-binding family protein	
AT4G28910	NINJA	Novel interactor of JAZ	
AT1G74950	TIFY10B	TIFY domain/Divergent CCT motif family protein	
AT1G70700	TIFY7	TIFY domain/Divergent CCT motif family protein	
**CLUSTER3**			
AT4G17500	ERF-1	Ethylene responsive element binding factor 1	
AT5G61600	ERF104	Ethylene response factor 104	Fungi
AT5G47230	ERF5	Ethylene responsive element binding factor 5	
AT4G17490	ERF6	Ethylene responsive element binding factor 6	
AT4G26750	EXT-like	Hydroxyproline-rich glycoprotein family protein	Fungi, bacteria
AT3G45640	MPK3	Mitogen-activated protein kinase 3	Bacteria
AT3G25882	NIMIN-2	NIM1-interacting 2	
AT2G42010	PLDBETA1	Phospholipase D beta 1	Fungi, bacteria
AT1G43700	VIP1	VIRE2-interacting protein 1	Fungi
AT4G31550	WRKY11	WRKY DNA-binding protein 11	Bacteria
AT4G31800	WRKY18	WRKY DNA-binding protein 18	Fungi, bacteria, virus
AT2G38470	WRKY33	WRKY DNA-binding protein 33	Fungi, bacteria
AT1G80840	WRKY40	WRKY DNA-binding protein 40	Fungi, bacteria, virus
AT2G25000	WRKY60	WRKY DNA-binding protein 60	Fungi, bacteria
AT3G56400	WRKY70	WRKY DNA-binding protein 70	Fungi, bacteria

In addition to the functional analysis based on the pathogen-specific defense pathway, we analyzed the retrieved proteins according to their functional association using the STRING web server. The obtained network was submitted to a clustering analysis through the Cytoscape app MCL cluster algorithm. Using a granularity parameter (inflation value) set to 2.5 with an edge weight cutoff to zero, we obtained 3 differentiated clusters (Figure 3C). Cluster 1 is composed of 30 proteins enriched in components of the defense response to bacteria, viruses, and fungi. This cluster also includes 3 RNA-binding proteins, which contain RNA recognition motifs and are proposed to stimulate ethylene production (UBA2A, At3g15015, and At2g41060; Kim C. Y. et al., 2008). Cluster 1 also comprises five isoforms of the heat shock protein 70 family, and it is enriched in members of the MOS4-associated complex (MAC; MAC3A, MAC5A, MLP423, MOS4). MAC is a highly conserved nuclear protein complex associated with the spliceosome with a role in RNA processing and/or splicing. The MAC founding member is MODIFIER OF SNC1, 4 (MOS4), identified in a suppressor screening of the gain of function mutant *snc1*. The *mos4-1* mutant could completely suppress all autoimmune phenotypes of *snc1* (Monaghan et al., 2010; Johnson et al., 2011). MOS2, the other suppressor of *snc1*, (Zhang et al., 2005) is also a candidate of SUMO modification.

Cluster 2 and cluster 3 are functionally closer in comparison to cluster 1. Cluster 2 with 16 members is enriched in JAZ transcriptional repressors and ethylene biosynthetic (ACO) and response (ERF) genes. While cluster 3, with 13 members, is enriched in ethylene response factors ERF and WRKY transcription factors, which include all 3 members of the group II-a (AtWRKY18, AtWRKY40, and AtWRKY60), and one representative of the group I (AtWRKY33), group II-d (AtWRKY11), and group III (AtWRKY70; Eulgem et al., 2000). In a recent study, WRKY33 has been validated as a SUMO substrate and provided evidence of the role of SUMOylation in defense responses mediated by WRKY33, supporting the present *in silico* analysis. In infection experiments of Col-0 plants with *B. cinerea* spores, SUMOylation of WRKY33 correlates with WRKY33 accumulation after 16 h post-inoculation. Similar to *wrky33* mutants, the expression of the SUMOylation-deficient WRKY33 (3K/R) conferred higher susceptibility to *B. cinerea* in detached leaves assays, suggesting a crucial role of SUMOylation in WRKY33 activity and a positive role in defense against necrotrophic pathogens. In addition, SUMOylation enables the interaction of WRKY33 with MAPK3/6. Additional analyses identified a SIM in MPK3 that facilitates WRKY33-MAPK3 interactions, which results in WRKY33 phosphorylation. On the other hand, plants expressing the SUMOylation-deficient WRKY33 (3K/R) are more resistant to *P. syringae* DC3000, suggesting a model in which the SUMOylation state of WRKY33 will elicit a specific set of defense responses to respond to the attacking pathogen (Verma et al., 2021).

Similarly to WRKY33, further studies to validate the identified proteins as bona fide SUMO targets are required. Nonetheless, this analysis provides a comprehensive up-to-date list of potential SUMO conjugates with a role in defense responses, which has been validated by the confirmation of WRKY33 as a SUMO target. Functionally, these proteins are enriched in transcription factors involved in signaling of the three major plant defense-related hormones, SA, JA, and ethylene. It is noteworthy that some of the candidates are known to interact with validated SUMO targets, such as SNC1 and FLS2, suggesting a potential role of SUMOylation as a coordinator of defense components by contributing to the formation of supramolecular protein assemblies to mediate trade-offs between growth and defense.

Overall, SUMO plays versatile roles in plant-pathogen interactions, ranging from PAMP perception, activation of stress-responsive gene expression, and fine-tuning plant hormone homeostasis. However, its function in establishing plant resistance or susceptibility depends on the plant-invader combination, further emphasizing the fact that a transversal role of SUMO in pathogen defense does not exist.

## Concluding Remarks

Since its discovery, multiple studies have pointed to SUMOylation as a master regulator of crucial plant growth responses, but also as a key player in plant-pathogen interactions. In defense responses, the plant SUMO system has a dual role by interacting with pathogenic effectors and by regulating major components of the plant defense machinery. Depending on their lifestyle, pathogens may hijack or subvert the plant SUMOylation system to establish successful infections. From the foregoing studies discussed, it becomes apparent that viruses exploit the role of SUMOylation as a regulator of replication through a mechanism that involves the recruitment of the SUMO E2 conjugating enzyme by viral proteins. Instead, pathogenic bacteria, which do not depend on the plant replication machinery for proliferation, use effectors to manipulate plant hormone levels and defense components to suppress the defense response. The bacterial effectors identified are multifunctional proteins that contain a SUMO protease domain, ULP, and other functional domains conferring transcriptional regulation or protein-protein binding capabilities. Whether the main mechanism of action of the ULP domain aims to deplete global SUMOylation or it provides a strategy to recruit the effector to subcellular compartments enriched in SUMO conjugates is a major unanswered question. From the plant side, the dissection of the defense SUMOylome has been addressed by targeted and untargeted studies that have identified positive and negative regulators of plant immunity. These are located at different layers of the defense response and include membrane and intracellular receptors, signaling components, and transcriptional regulators, highlighting the complex role of SUMOylation in coordinating defense responses. Simplistic conclusions on the role of SUMOylation in plant defense are hampered not only by the complexity of plant-pathogen interactions, but also by the complexity of SUMOylation itself. While plant-pathogen interactions are highly susceptible to environmental conditions and the host and pathogen fitness, SUMOylation is a very labile PTM that can modulate the target through covalent and non-covalent interactions with unpredictable molecular consequences. In addition, the study of SUMOylation is technically challenging, requiring non-standardized biochemical approaches. As an alternative to the lack of robust analytical tools, heterologous systems are frequently used, which may have several limitations related to the specificity and *in vivo* relevance. Future technological advances aiming to monitor SUMOylation *in vivo* dynamics could greatly contribute to overcome these limitations and, to dissect the relevance of SUMO during the different stages of pathogen attack and its implications in plant survival. In crops, instead of focusing on specific SUMO targets, most of the performed studies have addressed the role of SUMO from a genomics perspective. The results obtained suggest that upregulation of SUMOylation genes constitutes a resistance trait. Interestingly, the SUMO E2 conjugating enzyme is the SUMOylation machinery component more frequently found in genomic studies, although the molecular mechanism supporting this pivotal role of SCE1 remains unknown. While further investigations are needed to elucidate the mechanistic insights into the regulation of defense responses by SUMO in crops, these results open a window of opportunity for engineering SUMOylation as an approach to improve pathogen resistance in crops.

## Author Contributions

All authors contributed to literature review and writing of the manuscript. DF was responsible for analysis of the SUMOylome.

## Conflict of Interest

The authors declare that the research was conducted in the absence of any commercial or financial relationships that could be construed as a potential conflict of interest.

## Publisher’s Note

All claims expressed in this article are solely those of the authors and do not necessarily represent those of their affiliated organizations, or those of the publisher, the editors and the reviewers. Any product that may be evaluated in this article, or claim that may be made by its manufacturer, is not guaranteed or endorsed by the publisher.
